# Family Policy and Child Well-Being: The Case of Montenegro in the European Perspective

**DOI:** 10.3390/ijerph18179118

**Published:** 2021-08-29

**Authors:** Branko Bošković, Harriet Churchill, Oriola Hamzallari

**Affiliations:** 1 Humanistic Studies, University of Donja Gorica, Oktoih 1, 81000 Podgorica, Montenegro; 2 Department of Sociological Studies, University of Sheffield, Elmfield Building, Northumberland Road, Sheffield S10 2TN, UK; h.churchill@sheffield.ac.uk; 3 Department of Psychology, Aleksander Moisiu University, Rruga Miqesia, Spitalle, 2000 Durres, Albania; oriolahamzallari@uamd.edu.al

**Keywords:** family, children, parents, well-being, Montenegro, European Union

## Abstract

Family policies and family support measures have been identified as having major implications for child well-being, particularly through their role in influencing parental and family resources, circumstances and behaviour. The official approach to family policies focuses on opportunities for families to balance their work and family duties and care for their children. This paper analyses the type of policies available in Montenegro compared to the European Union. Potentially, Montenegro will become an EU member state, thus it is important to take a look at Montenegrin practice, as children should have equal life chances and protection of their well-being. Having a solid legal framework per se does not necessarily result in significant positive outcomes, and this paper analyses whether children in Montenegro have the same opportunities for development, in the context of family policies, as their counterparts in the rest of Europe. The focus of the paper will be on the criteria that define family rights and obligations, eligibility, availability and use of family policies in Montenegro. Based on the specific measures and datasets examined, the analysis considers the degree to which a period of family policy investment in Montenegro has been accompanied by improvements in child well-being and family resources, and undertakes comparisons in these regards with EU-wide family policy and child well-being trends. The paper uses a welfare state theoretical approach, with the focus on social investment and relevant data on children’s well-being obtained from the Eurostat, the OECD and the official national statistics.

## 1. Introduction

Improving the well-being of children has deep and long-lasting benefits for societies. Researchers and policy makers alike have long investigated potential means of improving societal mechanisms aimed at children and families. This paper examines the advances made by Montenegro as a result of reforming its family policies. It does so by comparing these reforms, and their outcomes with wider EU trends and by considering the extent to which these changes in family policies go hand in hand with improvements in the well-being of children and families. As a recognised candidate for membership of the EU, Montenegro is progressively adopting family policies in accordance with EU standards and requirements. However, there is a pressing need for research investigating the extent to which reforms to family policy in Montenegro are in fact associated with improved conditions for children and families.

This paper analyses Montenegro’s major family policy documents and focuses on those documents recognised as directly linked with children’s well-being, such as parental leave policies and early education policies, and on research that investigates the well-being of children and families. These are then compared nationally and to wider EU trends. Using a macro-level comparison approach, the living conditions of children and families in Montenegro are evaluated more broadly. This reveals potential trends that may hinder, or support, the well-being of children and families.

There has been much theoretical work carried out with the aim of developing family policies. Reflecting the various social changes that have taken place over preceding decades, a wide variety of different approaches to family policies have emerged. Recent work on family policy has investigated changing childbearing and partnership trends, the effects of different household structures on childhood well-being, the relationship between well-being, economic activity and socio-economic trends, and the consequences of an ageing society [1]. Given the importance of childhood well-being, research into the effects of family policies must be innovative as well as empirically well-grounded. Considering systemic factors such as globalisation, migration, and common forms of economic crisis [2] reinforces the necessity of taking a systemic and coordinated approach to the well-being of families and children.

In what follows, family policies are taken to be “policies associated with families with children”, and as policies that seek to promote the functionality and well-being of families with children [3]. In Kamerman and Kahn’s [4] terms, the focus of this paper is explicit family policies. These are policies with the express purpose of achieving aims regarding the family, contrasting with policies that may incidentally have consequences for families and children, despite this not being their stated aim. Taking a proactive approach, this paper investigates and analyses policies which have as their aim the creation of a more equal and just society.

Although family households, parents’ circumstances and the well-being of children are the main focus of this paper’s analysis, ‘family’ is recognised as having multiple meanings and applications across different European contexts. It is important to recognise a diversity of family household formations, relationships and practices within and across countries, as well as the diverse ways in which the family lives of children and parents operate beyond and across households. Furthermore, this paper takes a critical perspective that challenges the inequalities and risks associated with family constructs and dynamics. The analysis presented here has as one of its background commitments that children’s rights, gender equality and social justice are crucial and necessary family policy goals [3,5].

The family policies explored and analysed in this paper are those that exist at the national level, created by national legislation and funded by a state’s central government. This is important to emphasize, as policy can also be established at subnational or supranational level and even at the level of individual private companies and organisations [3]. Additionally, family policy is not amongst the EU’s direct competences. Family policy therefore falls within the legislative purview of individual member states. Nevertheless, the significant number of policy recommendations made by the EU constitutes a solid legal framework.

The theoretical approach used in this paper is welfare state theory, with a particular focus on the social investment paradigm. This is a future-oriented approach which investigates the development of human capital and the opportunities for investing in citizens from the earliest age. The approach aims at prevention and preparation, developing proactive measures rather than acting after the fact [6]. According to the approach taken here, social policy is a precondition for economic growth [7]. The concept of social investment is used to analyse policies with the explicit goal of protecting human capital in parents and developing it in children. Family policies are understood as pivotal in virtue of the role they play in enabling family development and creating the best possible conditions for children, through high-quality preschool education and care institutions.

A study published by the OECD in 2009 demonstrates that family policies can have a positive impact on children’s well-being [8]. The study investigates the effects of cash transfers, parental pro-employment policies and in-kind services with different policy approaches. The study’s results demonstrate that increased family income has positive effects on children, as measured by cognitive ability and educational outcomes. The effect is particularly pronounced in early childhood. The study also demonstrates that early intervention for at-risk children has the most profound effect, due to a longer pay-off period, greater malleability of cognitive outcomes, and complementarity between spending in early and later childhood [8].

Other studies have produced similar results. Norwegian practice reveals the positive effects of universal preschool childcare and education on educational outcomes in high school and beyond, as well as on earnings [9,10]. Baker et al. [11] present a number of studies which also highlight positive outcomes of universal childcare programmes, especially for disadvantaged and at-risk children. Their study also finds that such childcare programmes have a positive impact on children’s educational aspirations later in life, e.g., in PISA test results [11]. A further study focusing on specific populations, in this case adopted children, also demonstrates the impact of parents’ socio-economic conditions on children’s educational outcomes [12].

One current debate is whether universal childcare for children older than three, offered in preschool education and care institutions, has an equally positive effect on all children. In other words, do all children benefit from this universal provision to the same extent or do outcomes vary depending on factors such as the economic status and welfare of parents, implying the need for a more targeted approach [13,14,15]? Considering this question is vitally important in virtue of the fact that preschool care and compulsory education represent a large investment of public funds and the impact of such policies should justify their expenditure. This, however, is not the only factor to consider. Parents’ responses to different policy approaches (e.g., availability vs. changes in childcare costs) also matter [13].

Javornik [16] argues that parental leave policies are of the highest importance for parents because of their potential to have negative consequences on women’s employment. Liu and Skans provide evidence that mothers being in employment leads to the creation of alternative human capital investments [17]. Given considerations such as these, the effective design of parental leave policies is important for both parents and children.

Familial and de-familial approaches both provide insights into the nature of caring obligations and the ways in which the design of a country’s welfare state reinforces one or the other [18,19]. The socialisation or marketization of family care either motivates the family to be the main provider of care or to find adequate caring services on the market, provided by either public or private institutions. In other words, the dominant approach taken in this policy area usually leads to one of two potential consequences. Taking one approach to the design of a country’s welfare state relieves the family of its caring obligations, allowing the continuation of work. This is thought to be of greater relevance to women. Alternatively, a country’s welfare state is designed in such a way that it prevents a return to work and provides other direct or indirect benefits for parents who carry out caring duties.

Normative assumptions about gender roles and the social organisation of care play a role in defining regulations dealing with parental leave and childcare services, a process referred to as policy conceptual logic [16]. The specifics of policy design are of crucial importance to the state because such decisions define the degree to which the state supports the dual-earner model of the family, representing the transformative potential of policy [16]. Family policies which impose conditions on parental leave may constrain the choices that families make in relation to children, especially if high-quality childcare provision is not available [16]. The potential for negative impacts on women means that decisions on parental leave policies must be made carefully. As a general pattern, mothers take periods of leave with greater frequency and length. This potentially undermines their opportunities for career progression, in turn leading to depletion of skills, lower wages, reduced access to more senior positions and negative external perceptions of their commitment to their job [20,21,22,23,24].

A child’s living conditions also influence the level of well-being and subsequent life opportunities. Poverty and lower family income may have a substantial negative impact on a child’s well-being. Additionally, the timing of poor or worsening living conditions has also been shown to be important, with the most negative effects being observed during a child’s preschool and early school years [25,26,27,28,29,30,31]. The negative impacts of poverty can be observed in educational outcomes [32]. Fernandez and Ramia document the negative effects of poverty on a child’s experiences at school, both socially and academically, and demonstrate that these effects are mostly indirect [33]. This research suggests that living conditions can significantly impact many different aspects of child’s well-being, despite focusing primarily on a child’s cognitive and non-cognitive abilities and achievements. Persistent poverty, as well as extreme poverty, appears to have a particularly negative impact.

## 2. Materials and Methods

The key materials reviewed and analysed in this paper are EU and OECD statistics, along with national and EU family policy documents and strategies. The paper reviews these official data and policy sources in order to evaluate recent reforms and trends in the particular case of Montenegro and compares these with prominent reforms and trends in the EU more widely. The EU and OECD statistics, drawn from Eurostat and the OECD family database, constitute the most comprehensive sources of official national statistics for European countries and provide the best basis for a comparative study. The paper also draws extensively on the European Union Statistics on Income and Living Conditions (EU-SILC), a major source of EU official data from 2003 onwards. This database provides longitudinal comparative national socio-economic data for EU member states, as well as for European countries that are not member states, covering multiple social and economic indicators and domains.

It is important to recognise that these data sources at best indicate associations between national reforms and national trends. For the purpose of policy evaluation, these associations require further empirical investigation. Nevertheless, the data provide valuable statistics that reveal cross-national and regional European trends which have been subject to close attention and have led to quality improvements. These data sources, however, also have limitations. For example, the sources often rely on individual and household statistics and on units of analysis which can only provide a partial picture of family membership, resources and dynamics [5]. The paper therefore takes a critical approach to the data analysed. It also examines key findings in light of additional national data and comparative studies. The review of family policy reforms at both the national and EU level considers the key features of national policy measures, including their goals, instruments, funding and implementation [3].

## 3. Results

### 3.1. Family Policies in Montenegro

Family policies have long been recognised in Montenegro’s legal framework. The Family Law, the Labour Law and the Law on Social and Child Protection set out the major policies in this area and how they can be implemented. The Family Law prescribes parents’ responsibilities and their duty of care towards children. This law ensures that children’s rights are guaranteed and protected. The same Law provides the rights and well-being of children and a high level of protection, clearly defining the role of the parents and ensuring the necessary institutional support. Parents are legally recognised as their children’s guardians. Any type of discrimination against children is forbidden and public institutions are legally obliged to care for children when their family is unable to [34].

The Labour Law and the Law on Social and Child Protection set out additional family policies as well as their availability and the criteria governing eligibility. The Labour Law ensures the provision of 365 days of parental leave, available for parents to use equally, of which 30 days are transferable [35]. The Law on Social and Child Protection sets replacement parental pay at 100% of the parent’s average income over the previous 12 months [36]. Disabled children are recognised as a vulnerable group and affirmative action policies are put in place for their benefit. These policies cover areas including availability of placement, transport and education, all of which are free during the period in which disabled children are in school. Further benefits are also afforded but are not discussed here due to cross-national incompatibility and limitations on space.

A second aspect of Montenegro’s family policies aims at supporting parents’ labour market participation. The policies in question concern preschool education institutions and the country’s care infrastructure, and the availability, accessibility and affordability of these resources. The EU’s Work–Life Balance Directive for working parents and carers recognises this as a crucial element in the ability of parents to remain part of the labour market [37]. Access to preschool education and to care institutions in Montenegro are free and available to all children, irrespective of their circumstances. Moreover, from 2019 onwards, all children enrolled in public preschool education have been guaranteed a place, again free of at the point of use. Private institutions are also available, but their prices vary, and the number of children enrolled in private institutions is around 5% of the total number of children enrolled in preschool education [38]. We can conclude from this that public preschool education and care are affordable to all of Montenegro’s citizens.

However, this policy has resulted in an increase in the number of children enrolled without a corresponding increase in capacity, resulting in class sizes as large as 70 in cities and urban areas [39]. This is a malfunction of what was supposed to be a positive policy intervention for parents and has had a having particularly negative impact in the most populous areas. These unintended outcomes raise questions about the quality of care and education provided to children. They also hinder the potentially positive outcomes of the increase in the participation rate of children in preschool education and care. The availability of such resources has to be planned in accordance with the actual capacities of preschool education institutions, something lacking in the Montenegrin case.

### 3.2. Family Policies in the Strategic Framework in Montenegro

Children’s rights and the features that constitute an appropriate upbringing are recognised in the Montenegrin strategic framework. The current strategic framework includes the Strategy for Exercising the Rights of the Child 2019–2023, the Strategy for Early and Preschool Education in Montenegro 2016–2020, the Strategy for the Prevention and Protection of Children Against Violence 2017–2021, the Strategy for Employment and Human Resources Development 2016–2020, the Inclusive Education Strategy 2019–2025, the Strategy for the Development of the Social and Child Protection System from 2018 to 2022 and the National Strategy for Sustainable Development until 2030. The scope of this paper does not allow for detailed individual discussion of these strategies. Instead, considering the paper’s primary concern, they are analysed in the context of children and family policies.

An analysis of the strategic framework reveals a lack of recognition of the importance of family policies in the context of children’s development and their well-being. Indeed, family policies are not even mentioned, other than in the National Strategy for Sustainable Development until 2030 [40]. This strategy recognises the problematic lack of family policies and stresses the need in an ageing society to increase the country’s birth rate at the same time as increasing women’s access to work. Therefore, in the context of this paper’s focus, family policies are not adequately recognised in Montenegro’s major strategic documents. Nevertheless, the strategic documents do set out the rights of children and clearly delineate the relevant stakeholders. Advancing, protecting and improving children’s well-being are all key goals of the Montenegrin government.

### 3.3. European Union and Family Policies: Recognition and Relevance

The European Union has recognised the importance of family policies and has placed them high on its social policy agenda. The EU Pillar of Social Rights affirms a child’s right to have an affordable early childhood education and good quality care; it also affirms that children should be protected from poverty and should have equal opportunities, especially those from disadvantaged backgrounds. The EU Recommendation on Investing in Children also provides firm guidance and policy recommendations seeking to improve children’s well-being and their future opportunities by stressing the need to invest in their human capital from an early age [41]. The Recommendation also points towards policies which aim at supporting the ability of parents to successfully balance work and family life [41].

A more recent and legally binding document, the Directive on Work–Life Balance for Parents and Carers [37], points firmly to the problems faced by parents, demonstrating the impact of specific policies on different family forms, most crucially in the long term. There is a need for a novel approach with special attention given to women’s employment and their working arrangements, to carefully designed leave policies and to the availability, accessibility and affordability of childcare [37]. The Feasibility Study on the Child Guarantee provides recommendations on how to improve opportunities for children from disadvantaged backgrounds [42]. The EU Youth Strategy 2019–2027 points to the three major principles governing EU action regarding the young: engage, connect and empower [43]. The Strategy also reveals the need for further improvement and for a formal approach to addressing the social conditions of the young, especially by better coordinating of policies and policy expenditure. The Strategy’s importance lies in its stressing of the necessity of providing adequate conditions for children and youth development.

These documents show the EU’s firm dedication to its long-term goals and reflect a careful approach to children and their development. Furthermore, the EU’s strategic planning includes a recognition of parents as an irreplaceable component of childhood development. Parents’ well-being is understood as a precondition for children’s well-being. This acts to reinforce the fact that family policies must be coordinated among all of the institutions which are part of the process.

### 3.4. What Does the Data Tell Us about Families and Children in Montenegro?

According to the EU-SILC data, the risk of poverty or social exclusion for the population as a whole was 30.5% in 2019, which is a decrease of 0.9% compared to the previous year [44]. The risk of poverty or social exclusion for children aged 0–17 decreased from 38.4% in 2018 to 39.4% in 2019, while the increase for the age group 18–24 was 1.4%, from 34.9% in 2018 to 36.3% in 2019. In 2019 the risk for single-parent families was 58.3%, an increase of 9.1% from 2018. The figures for two-parent families are 34.4.% for 2018 and 33.5% for 2019 [44]. The rate is similar for men and women, 30.5%, which is a decrease for both groups compared to 2018. The data is presented in the Table 1.

A comparison of living conditions in Montenegro with the EU reveals significantly higher rates of risk of poverty and social exclusion in Montenegro than in the majority of member states, as well as the EU averages. Compared to Montenegro, the risk of poverty and social exclusion (AROPE) was higher in 2019 in Bulgaria (32.8%) and Romania (31.2%), and similar in Greece (30%). However, the standard deviation for AROPE for the EU in 2019 is 5.25, which reveals strikingly large differences among countries. The risk for children up to 17 years of age was also higher in Montenegro in 2019 when compared to the EU average (23.4%). It was also higher than in any EU member state, the closest being Romania (35.8%), Bulgaria (34.1%), Greece (30.5%) and Spain (30.3%) [44]. Similarly, differences among the EU countries are large, with the standard deviation being 6.43.

Single-parent families in 2019 faced a higher risk of poverty or social exclusion in Montenegro (58.3%) than the EU average (43%) [44]. The same percentage (58.3%) of families facing this risk was observed in Ireland, while the percentage stood at 51.6% in Bulgaria, 51.4% in Greece, 51.3% in Malta and below 50% in all other member states [44]. The standard deviation for the EU was high for this indicator as well (6.82), again revealing large differences between EU member states. The majority of EU member states saw a decrease in the level of risk for single-parent families. This contrasts with an increase in this indicator in Montenegro, from 49.4% in 2017 to 58.3% in 2018 [44]. Two-parent families in Montenegro are facing a comparatively higher risk of poverty and social exclusion than their counterparts in the EU, with the level of risk standing at 33.5% in 2019, compared to the EU average of 18.7%. Once again, however, there are significant variations among EU member states, reflected in a standard deviation of 6.48. When compared to Montenegro, similar percentages were observed in Romania (31.2%) and Greece (30.7%). Women in Montenegro are at risk to the same extent as men, recorded as 30.5% for both in 2019. However, when compared to the women in the EU, where the average is 22.3%, the risk level for women in Montenegro is almost 50% higher [44].

The level of participation of children aged between 4 and 6 in preschool education and care institutions has steadily increased in Montenegro since 2015. In 2015 a total of 16,972 children were enrolled, compared to 21,663 children in 2018 [45,46]. This is mainly the result of numerous campaigns, beginning in 2015, aimed at increasing the level of preschool enrolment. The preschool participation rate has increased from 58.66% in 2016 to 72.62% in 2018 [47]. Nevertheless, this is a significantly lower rate than the EU average of 95.4% [48]. The lowest numbers for the EU are observed in Slovakia (78.2%), Greece (81.5%) and Croatia (82.8%) [48].

In Montenegro, the average class size in 2019 was 29.7, with an average of 31.6 pupils in public institutions and 13.5 in private institutions. The pupil–teacher ratio was 16.6 on average, with 16.8 in public institutions and 13.8 in private institutions [38]. More than 95% of children attended public institutions in 2019. As previously mentioned, however, the reality is that there is a large difference in class sizes across the education system.

When considering the matter of which parent acts as the primary caretaker and therefore takes parental leave, we can note that the number of fathers taking paternal leave more than doubled between 2018 and 2019 from 203 to 421 [49]. However, this is still a very low number in relative terms, representing between 0.3% and 0.4% of fathers.

This low level of parental leave uptake by fathers, which can be safely assumed to have been significantly lower in 2015, can be compared with available data for the EU for 2015. When this comparison is carried out, the level is revealed to be well below the EU average of around 10% [50]. Once again, however, the numbers within the EU vary, from 0.02% in Greece to 44% in Sweden. In addition to Greece, the level was also under 3% in: Greece, Cyprus, Croatia, Slovakia, Lithuania, Austria, France and the Czech Republic [50].

The social protection budget in Montenegro has decreased in relative terms (Table 2), from 17.7% of GDP in 2017 to 16.6% in 2018 [44]. In 2018, the social protection budget when measured as a percentage of GDP was significantly lower in Montenegro (16.6%) than in the EU (26.7%), with a standard deviation of 5.68. The Montenegrin figures are similar to Ireland, Bulgaria, Romania, Malta, Latvia and Lithuania [44]. There is also see a disparity between Montenegro and the EU when looking at different indicators. For example, in Montenegro expenditure on family and children is equal to 4.2% of the total budget, compared to an EU average of 8.32% [51].

The OECD data reveals large differences in replacement parental pay, when measured as a percentage of previous earnings [52]. The data for the EU for 2015 shows that the average replacement rate in Eastern European countries was 86% and 93% (Table 3) in other member states [53]. Montenegro fares well both in this regard as well as with regard to the length of parental leave, offering a greater amount of leave than most EU member states. The average maternity leave in Eastern European countries was 27 weeks and 20.4 weeks in other member states [53]. Montenegrin parents are offered 54 weeks of leave, around double the EU average.

## 4. Discussion

An overview of the Montenegrin implementation of family policies reveals a solid legal framework which is not reflected in similarly positive outcomes. Nor, it must be admitted, is there a strategic recognition of family policies. In comparison with EU member states, Montenegro is lagging behind in practice. This is despite, as could be seen in the previous section, the more favourable legal requirements and conditions with regard to at least some family policies.

The analysis carried out in the previous section demonstrated significantly higher risks for all of the social groups relevant for our purposes, particularly for single-parent families. This is all the more pressing given that single-parent families are not recognised in the Montenegrin strategic framework as being vulnerable or at a higher level of risk. Data shows that the yearly number of divorces has steadily increased between 2016 and 2019, from 703 to 841 [54]. This points to an additional degree of risk, following an increased number of prospective single-parent families. Single-parent families face a higher risk of poverty and social exclusion when compared to two-parent families, with the level of risk increasing in 2019. Two-parent families offer a greater degree of security for the children raised in them. The fact that the number of single parents is likely to be increasing, together with the attendant increased level of risk, reinforces the crucial importance of this issue. Despite this, its importance is still not reflected in Montenegro’s actions regarding families. In the long run, this failure could lead to severe reductions in the level of well-being of children raised in single-parent families. Overall, the living conditions of Montenegrin citizens are similar to the worst-performing EU member states. On most metrics, Montenegro fares even more poorly than these member states and in all cases fares significantly worse than the EU average.

The ample provision of maternity leave in Montenegro, especially in comparison to EU member states, provides an opportunity for women to take long and well-paid breaks from their work. However, this provision also leaves unanswered questions concerning women’s future prospects and long-term work opportunities, which may be negatively impacted by a long absence from work. Shortening the statutory period of leave, however, is unlikely to have a positive impact on the well-being of either women or, especially, children. Such a change would raise concerns about the quality of care that mothers are able to provide and may lead to periods of leave being taken by other means. Single parents face an additional risk as they need to successfully combine work and sole caring responsibilities. The provision of long periods of parental leave can be expected to have a positive impact on children’s well-being.

The 2019 Eurydice report includes Montenegro in its comparison of early childhood education and care (ECEC) standards and aligns Montenegro with the highest ranked countries in the EU. One criterion the report uses to assess countries is the educational requirements placed on staff working with children. Montenegrin laws require staff to have a bachelor’s degree or higher, which is the case in one third of the countries included in the report [48]. Montenegrin laws also require assistants to have an initial qualification. However, there is no requirement for staff working in ECEC to undergo continuing personal development, which would be a step towards increasing standards. Comparatively speaking, however, the majority of the EU member states have similar requirements. Finally, on the criteria of the type of settings (unitary or separate), having one or more authorities overlooking ECEC, staff qualifications and education guidelines, Montenegrin ECEC is labelled as integrated [48]. From this it can be inferred that early education and care standards in Montenegro have been increased to equal the highest standards found in its European neighbours.

Nevertheless, keeping in mind the number of children in classrooms, as well as inadequate knowledge and skills testing of practitioners [55], the actual consequences for children’s well-being and education might not be as straightforwardly positive as the statutory requirements would suggest. As a consequence, it is doubtful that Montenegro’s approach to children’s well-being results in the best possible outcomes. A lack of studies in this policy area hinders drawing further conclusions. Implementation of strict criteria for evaluating the work of practitioners would provide data with which further conclusions could be drawn.

Educational outcomes can also be understood as consequences of a high-quality family policies framework. For the purpose of this paper, PISA test results are considered. With regards to average test results, in 2018 Montenegro was placed 54th out of 77 countries, with an average score of 422 [56]. It was well below the highest-ranking EU countries (Estonia with an average score of 525.3 and Finland with 516.3) and close to Bulgaria (426.7), Romania (428) and Cyprus (438) [56]. Results are similar for the three test categories (mathematics, science, reading), where Montenegrin pupils score significantly lower than pupils in EU member states. It must be stated, however, that these results are not solely a result of family policies but also of the broader features of the educational system and the quality of education provided within it.

The use of preschool education and care in Montenegro has only increased significantly in the last decade or so, a consequence of the familial policy approach taken during the previous period. During this period, families were expected to care for children at least until the age of 3, or a year before the child began attending school. Montenegro also has a long history of men being expected to be their family’s breadwinner. This has started to change, especially in the last two decades, bringing Montenegro in line with most of the countries that belong to what Esping Andersen calls the conservative model of the welfare state [57,58]. The fact that this can only partly explain the situation that Montenegro finds itself in suggests the necessity of appealing to other factors, such as historical and cultural traits. Montenegro’s strategic framework aims at improving educational opportunities for children but the potential of family friendly policies to achieve this aim has not yet been recognised. This is particularly pronounced when it comes to decreasing overall inequality among children, given that family friendly policies have a great deal of potential in this area.

A comparatively lower level of paternal leave uptake, together with a lower level of childcare participation, jointly imply that women carry a greater childcare burden in Montenegro than women in EU member states. In the long term, this may create pressures on the economic stability of families, subsequently endangering children’s equal access to opportunities. Lingering questions over the quality of childcare, as a result of overcrowded classes and practitioners being insufficiently knowledgeable and poorly tested, provide additional pressure on families, especially on single parents.

The data on the social protection budget shows that Montenegro spends less on cash and in-kind benefits related to households than the average EU member state [59]. Although this is a crude indicator, it is nevertheless instructive that the amount is lower both as a percentage of the social protection budget and as a percentage of GDP. Other indicators suggest similar conclusions. One exception is the “survivors” budget. However, it cannot be expected that this group of benefits can compensate for a lack of spending on families and children.

These conclusions demonstrate the need to further investigate the systemic causes of the poor outcomes of what is currently a very de-familial policy approach. This is an approach which is supportive for mothers and creates positive conditions for childcare enrolment. However, the case of Montenegro reveals that the well-being of children requires the careful coordination of different policies, including, but moving beyond, family policies. A lack of strategic recognition of family policies strongly indicates that Montenegro lacks a future-oriented approach. This would be one that takes advantage of the cumulative positive outcomes of family policies. Instead, Montenegro has individual policies which serve their purpose of providing rights to citizens but fail to create conditions in which these citizens can prosper. A longitudinal study on educational and occupational attainment would allow for more nuanced results and would provide further, and more detailed, insights.

Montenegro’s status as a merely prospective EU member may leave those citizens who are now children with significantly lower opportunities for a prosperous life. This is because their human capital has not been optimally developed. Montenegrin children live in worse social conditions than children in the EU. As a result of this, as well as other negative outcomes such as a worse quality of care in preschool educational institutions, their well-being is lower than those living in EU member states.

Single-parent families are facing an increased risk of poverty and social exclusion. Together with the lower quality of care that may result from increased class sizes in preschool education institutions, the well-being of these children is severely endangered. PISA test results for Montenegro’s current high school students reinforce the need to address these issues by planning policies in accordance with the other relevant policy areas. Furthermore, policies need to be planned in a way that their joint outcome does not undermine the intended positive consequences of any individual policy. Vulnerable groups must be clearly defined, as must those policies which aim at promoting their well-being. In the context of family policies, this means special attention must be paid to policies aimed at single parents.

## 5. Conclusions

This paper examined family policies in Montenegro and compared them with those in the European Union. It has been demonstrated that the Montenegrin legal framework takes a positive approach to family policies with regards to the duration of parental leave, as well as to the replacement rate of parental pay, which is higher than the EU average. Furthermore, access to a preschool education and to care institutions is provided to all children, allowing parents an opportunity to work. However, evaluation of the data shows that the relevant indicators lag behind the positive intent behind the legal framework. As a result, when compared to those living in EU member states, children and parents in Montenegro enjoy significantly lower living standards and face a higher risk of poverty and social exclusion.

Despite the favourable conditions created by generous parental leave and the universal provision of preschool education and care, single-parent families face a higher risk of poverty and social exclusion. The analysis carried out here demonstrates that children in Montenegro have lower levels of educational attainment and, together with the higher risks they face, that their well-being is placed under significantly more strain than that of their EU counterparts. Similarly, the human capital of Montenegrin children may not be as fully developed as that of children in the EU. These conclusions demonstrate the need to reconsider the implementation of these policies and the necessity of further policy coordination. Successfully doing so has the potential of bringing Montenegro in line with the EU and decreasing the level of risk faced by its children.

## Figures and Tables

**Table 1:** Risk of poverty or social exclusion in Montenegro and the European Union.

Category	2018	2019	2018	2019
	Montenegro		European Union	
Total	31.4	30.5	21.8	21.4
0–17	39.4	38.4	24.2	23.4
18–24	34.9	36.3	28.5	28.1
Men	31.7	30.5	20.8	20.4
Women	31.1	30.5	22.8	22.3
Single parent families	49.4	58.3	45	43
Two-parent families	34.4	33.5	20	19.5

**Table 2:** Social protection budget in Montenegro and the European Union.

Social Protection Budget	2018	2018
	Montenegro	European Union
Total	16.6	27.9
Percentage for the function “children and families “	4.2	8.32

**Table 3:** Replacement rates for parental and average length of maternal leaves in 2015.

Category	Montenegro	Eastern EU Countries	Non-Eastern EU Countries
Average replacement rate for parental leave	100%	86%	93%
Average length of maternity leave (weeks)	54	27	20.4

## Data Availability

Publicly available datasets were analyzed in this study. This data can be found here: https://ec.europa.eu/eurostat/data/database; https://www.monstat.org/eng/index.php (access on 7 July 2021); https://data.oecd.org/ (access on 7 July 2021).
